# Measurement model of credit risk for unlisted agricultural enterprises

**DOI:** 10.1371/journal.pone.0332124

**Published:** 2025-09-25

**Authors:** Kaihao Liang, Yuqiu Chen, Tinghong Guo, Tieshan He

**Affiliations:** 1 Department of Mathematics, Zhongkai University of Agriculture and Engineering, Guangzhou, Guangdong, China; 2 College of Economics and Trade, Zhongkai University of Agriculture and Engineering, Guangzhou, Guangdong, China; Buckinghamshire New University – High Wycombe Campus: Buckinghamshire New University, UNITED KINGDOM OF GREAT BRITAIN AND NORTHERN IRELAND

## Abstract

This paper aims to measure credit risks of unlisted agricultural enterprises by using the KMV model integrating a CNN-BiLSTM neural network. Initially, the expected default frequencies (*EDF*) for each listed agricultural enterprise are computed using the Black-Scholes option pricing formula within the KMV framework. We apply the neural network model trained by listed agricultural enterprises to the credit risk analysis of unlisted agricultural enterprises. The *EDF* and financial data of listed agricultural enterprises undergo Z-score standardization and comparison using CNN-BiLSTM neural networks. Model parameters are then experimented with to determine the optimal CNN-BiLSTM model. This selected optimal CNN-BiLSTM model is applied to standardized financial data of unlisted agricultural enterprises to derive corresponding *EDF*. Based on the *EDF* of the listed agricultural enterprises, corresponding rating intervals are determined for unlisted agricultural enterprises. We use unlisted companies in China as an example in empirical analysis. The results demonstrate the effective assessment of credit ratings for unlisted agricultural enterprises using this model, generally aligning with institutional rating outcomes. Given differences in rating systems, the model helps identify hidden credit risks that are challenging to detect through conventional rating methods. It highlights the nonlinear relationship between enterprise credit risks and financial indicators, including debt repayment capacity, operational capability, growth potential, profitability, and debt structure.

## 1 Introduction

Agricultural enterprises face higher credit risks compared with listed companies. Moreover, due to the limited data, it is difficult to assess the default risk of non-listed agricultural enterprises, which increases the risk in the financial market. Establishing a dependable risk assessment system is especially crucial for vulnerable sectors such as agriculture.

This research enhances the current KMV model to improve its relevance, especially for non-listed agricultural companies. Through the integration of machine learning technology, we establish a model that can accurately measure the default probability for agricultural non-listed enterprises and establish a framework that links expected default frequencies (*EDF*) with credit ratings.By merging the features of CNN-BiLSTM neural networks with the KMV model, this study expands the scope of the KMV model in assessing credit risks of non-listed agricultural enterprises. Theoretically, this study develops the risk measurement model from KMV and BP-KMV to the CNN-BiLSTM-KMV method. Practically, the method of combining CNN-BiLSTM and KMV model can effectively assess the credit ratings of non-listed agricultural enterprises, identifies the hidden credit risks that traditional methods are difficult to detect, and provides accurate risk suggestion for financial institutions.

This study utilizes various models, including credit metrics, credit portfolio view, and KMV, to measure and predict the credit risk of unlisted agricultural companies. The improved KMV model with widespread application [1], utilizes publicly available capital market data to measure default probabilities. Recent research by Zhang (2022) and Yang et al. (2022)[2] has validated the robust predictive ability of the KMV model in identifying credit risks, emphasizing that a shorter default distance (*DD*) is indicative of higher risks. Deep learning models are commonly used for financial risk prediction and early warning of listed enterprises [3]. We combined the KMV model with deep learning to measure the credit risk of non-listed agricultural enterprises. The motivation is that, the credit risk of agricultural enterprises is relatively high, and for non-listed agricultural enterprises, due to limited data, their default risk is difficult to assess, which increases the risk in the financial market. The KMV model integrated with CNN-BiLSTM neural network can identify the hidden credit risk of non-listed agricultural enterprises.

However, non-listed enterprises lack equity market information, which limits the application of the KMV model. Addressing this issue requires accurate collection and utilization of corporate financial data for risk assessment. The commercial confidential KMV’s PFM model for non-listed enterprises restricts its dissemination and usage due to confidentiality constraints. The BP-KMV model proposed by Zeng et al. (2017) introduces a novel method for implementing the KMV model in non-listed companies [4]. Through neural network techniques, it effectively assesses the credit status of non-listed companies, closely aligning with actual conditions. These studies indicate that combining neural networks with the KMV model can effectively address credit risk assessment issues for non-listed enterprises.

In recent years, scholars have made significant advancements in credit rating by utilizing machine learning and neural network methodologies. Chen et al. (2020) [5] proposed the SMAGRU model based on recurrent neural networks. This model assigns market benchmark weights by capturing time series features, demonstrating outstanding performance in multi-class credit rating classification. Cai et al. (2020) [6] applied decision tree algorithms and binomial logistic regression to improve the accuracy of credit risk assessment in P2P network lending, thereby reducing platform loan risks. Dastile et al. (2020) [7] introduced a machine learning framework, and demonstrated that classifier ensembles are more effective than individual classifiers. Tsai (2010) [8] explored the application of deep learning models in credit rating, achieving outstanding predictive accuracy and profitability through various model combinations. Shen et al. (2021) [9] combined LSTM networks with the AdaBoost algorithm to develop a deep learning model for managing imbalanced credit data, demonstrating competitiveness in credit risk assessment. Guo et al. (2019) [10] proposed a multi-level adaptive classifier ensemble model, enhancing the performance and adaptability of credit risk models by utilizing data preprocessing and parameter selection based on Bayesian optimization. Zhao et al. (2025) [11]introduced a novel method for credit scoring of retail bank customers using deep learning techniques and demonstrated its effectiveness. Fama et al. (2024) [12] proposed an explainable machine learning method for financial risk. Gotze et al. (2020) [13] compared enhanced machine learning models with linear regression models and found that random forests outperformed them in predictive performance of credit risk. Zhou et al. (2019) [14] proposed a large-scale data mining method based on particle swarm optimization for backpropagation neural networks. This method is characterized by fast convergence and strong predictive capability. Pang (2021) [15] introduced an assessment model for evaluating borrower credit level by employing extreme learning machines and fuzzy algorithms. Wen (2021) [16] optimized the random forest model using particle swarm optimization to reduce error rates in bank credit risk assessment. Khan et al. (2021) [17] presented a CNN-based method that achieved superior rating prediction outcomes. Li et al. (2021) [18] enhanced the optimal partition algorithm and RBF neural network structure to improve the robustness and accuracy of credit risk models. Li and Chen (2025) [19] utilized multi-source data combined with random forest to effectively assess the debt risk of local government financing platforms. Hou et al. (2024) [20] proposed an improved search algorithm to optimize the LightGBM model, providing a new solution for credit risk prediction of small and medium-sized enterprises in supply chain finance. Zhu and Wu (2025) [21] applied graph neural networks to P2P credit risk management, expanding the application of graph models in this field. Kwon et al. (2025) [22] employed multi-task twin neural networks for credit scoring, enhancing the prediction performance while improving the model stability.

These studies demonstrate the extensive application and innovation of machine learning and neural networks in the field of credit rating, offering new methods and perspectives to improve the accuracy and efficiency of credit assessment.

The main contribution of this study lies in integrating the KMV model with the CNN-BiLSTM neural network to construct a credit risk measurement model for non-listed agricultural enterprises, addressing the issue of the lack of equity data for non-listed enterprises, effectively assessing their credit ratings, and identifying hidden credit risks that traditional methods may fail to detect. CNN is used to extract local features of multi-source data such as financial and operating indicators. BiLSTM is employed to capture temporal dependencies, thereby supplementing non-structured information and dynamic changes for KMV [11]. Compared with the pure KMV, the combination of KMV and BiLSTM breaks through the limitation of relying solely on stocks and financial data, which can promptly respond to market anomalies, avoid distribution assumption deviations. Compared with the BP-KMV model, the combination of the KMV model and CNN-BiLSTM can more comprehensively excavate the nonlinear, multi-scale correlations and temporal dynamic patterns in the data, effectively overcoming the limitations of the BP network in modeling long sequence dependencies [1].

The remaining part of the paper is organized as follows: Sect 2 introduces the credit risk evaluation index system for non-listed agricultural enterprises. In Sect 3, the calculation method of expected default frequency in the KMV model is elaborated. Then, in Sect 4, the principles, frameworks, and applications of convolutional neural networks and bidirectional long short-term memory networks in EDF prediction are presented. Sect 5 is the empirical analysis part, covering data sources and standardization, model construction, training, parameter comparison, optimization, and credit rating. Sect 6 presents the conclusion of the study.

Notations: *EDF* denotes the expected default frequency of a company, *DD* represents the default distance of an enterprise. N(·) is the distribution function of the standard normal distribution. Suppose that *A* and *B* are matrices, *A* * *B* denotes the convolutional product of *A* and *B*.

Abbreviations: CNN:onvolutional neural networks; BiLSTM: bidirectional long short-term memory networks; ReLU: rectified linear unit; ROE: return on equity; ROA: Return on assets; MAE: mean absolute error; RMSE: root mean square error; MSE: mean squared error; MAPE: mean absolute percentage error.

## 2 Credit risk evaluation indexes of unlisted agricultural companies

This study combines relevant financial indicators to construct a default risk evaluation system for non-listed agricultural enterprises. Due to their smaller scale, non-listed agricultural enterprises are relatively less affected by fiscal taxation and macroeconomic factors compared to large listed agricultural enterprises. Therefore, only financial data from non-listed enterprises are analyzed and summarized into 12 financial indicators across 5 categories: profitability level (C1), debt repayment level (C2), operational level (C3), debt structure (C4), and growth level (C5). The selected indicators fully reflect the comprehensive financial situation of enterprises. Specific details of the evaluation index system are presented in Table 1. The financial indicators utilized in this study cover various aspects of company performance and financial health. For profitability (C1), metrics such as return on equity (ROE) and return on assets (ROA) assess the efficiency of profit generation relative to shareholder equity and total assets, respectively. Debt repayment capacity (C2) is evaluated through indicators such as the current ratio and quick ratio, which reflect the company’s ability to repay short-term debts using current and quick assets. Operational efficiency (C3) is measured by the inventory turnover ratio and current asset turnover ratio, reflecting the company’s inventory management and asset utilization efficiency. Debt structure (C4) indicators, such as the financial liability ratio and debt-to-asset ratio, provide insights into the company’s leverage and capital structure. Growth capability (C5) metrics, such as the operating income growth rate and net profit growth rate, assess the company’s growth potential and operational performance. ROE and ROA reflect the efficiency of profit-making, the current ratio indicates the ability to repay debts, and the inventory turnover rate measures operational efficiency. Natural risks may lead to reduced agricultural production or deterioration in product quality, directly reducing operating income and causing a decrease in ROE. The occurrence of natural risks will result in depreciation of inventory and fixed assets, thus causing an increase in the financial liability ratio. Policies have a multi-directional impact on indicators by increasing income, reducing costs, and optimizing financing [23]. For example, low-interest loans or fiscal subsidies supported by policies for purchasing agricultural equipment will lead to an increase in ROA. The construction of policy-supported cold chain logistics reduces inventory losses and improves the inventory turnover rate. These indicators constitute a comprehensive evaluation of a non-listed agricultural company’s financial condition and performance.

**Table 1:** Specific financial indicators of non-listed agricultural companies.

Indicator category	Specific item	Symbol
Profitability (C1)	Return on equity (ROE)	*X*_1_
Profitability (C1)	Return on assets (ROA)	*X*_2_
Debt repayment (C2)	Current ratio	*X*_3_
Debt repayment (C2)	Quick ratio	*X*_4_
Operational efficiency (C3)	Inventory turnover ratio	*X*_5_
Operational efficiency (C3)	Current asset turnover ratio	*X*_6_
Debt structure (C4)	Financial liability ratio	*X*_7_
Debt structure (C4)	Debt-to-asset ratio	*X*_8_
Debt structure (C4)	Non-current liability ratio	*X*_9_
Growth capability (C5)	Operating income growth rate	*X*_10_
Growth capability (C5)	Net profit growth rate	*X*_11_
Growth capability (C5)	Operating profit growth rate	*X*_12_

## 3 Expected default frequencies with KMV model

Accurate assessment of credit risk requires quantifying the level of credit risk. This study uses the *EDF* from the KMV model to quantitatively evaluate the default risk of non-listed agricultural enterprises. Because of the absence of equity data for a non-listed enterprise, its *EDF* is estimated using neural networks trained on data from listed companies. Following the computational steps of the KMV model, the enterprise’s *DD* is calculated first based on the equity information of listed companies, risk-free rates, and short- and long-term debts. Subsequently, the *EDF* is derived based on the mapping relationship between *DD* and *EDF*.

First, the daily logarithmic returns $\mu_{i}$ and return volatility $\sigma_{D}$ are calculated based on the daily closing price *S*_*i*_ of listed companies, as shown in formulas (1) and (2), respectively. Then, the annualized return volatility $\sigma_{E}$ is computed by (3), where *i* represents the trading day index ranging from 1 to *n*, with *n* typically set to 252 days.

$$\mu_{i}=\ln(S_{i-1}/S_{i}),$$ (1)

$$\sigma_{D}=\sqrt{\frac{\sum_{i=1}^{n}(\mu_{i}-\bar{\mu }{)}^{2}}{n-1}},$$ (2)

$$\sigma_{E}=\sigma_{D}\times \sqrt{n}.$$ (3)

The Black-Scholes option pricing formula is utilized to calculate the *DD* in the KMV model. This model assumes continuous asset value and a normal distribution. The value of the asset of interest, treated as a call option, can be calculated using the formula (4),

$$DD=\left[\ln\left(\frac{V_{A}}{B}\right)+\left(r+\frac{\sigma_{A}^{2}}{2}\right)\right]/\sigma_{A}\sqrt{t},$$ (4)

where $V_{A}$ represents the asset value, *B* is the book value of liabilities, *r* is the risk-free interest rate, and *t* is the debt maturity.

This probability *EDF* is obtained by establishing a mapping relationship between the computed *DD* and the corresponding *EDF* in formula (5),

EDF=[1−N(DD)]×100%, (5)

where N(·) is the distribution function of the standard normal distribution.

## 4 CNN-BiLSTM neural network model

### 4.1 Convolutional neural networks

The CNN-BiLSTM neural network model combines convolutional neural networks (CNN) with bidirectional long short-term memory networks (BiLSTM) to effectively process sequential data containing spatial features and temporal dependencies. The convolutional layer, as proposed by Li et al. (2016) [18], constitutes a critical part of CNNs. Convolutional neural networks extract hidden features from input data using convolution and pooling operations, merging these features before feeding them into fully connected layers. In this process, each convolutional layer contains multiple filters that capture hidden features from the input and generate feature maps. The output of the convolutional layer undergoes nonlinear activation functions, with common choices including rectified linear unit (ReLU). Mathematically, convolutional operations can be represented as follows:


$$c_{i}=f(w_{i}\;*\;x_{i}+b_{i}),$$

where *x*_*i*_ represents the input to the convolutional layer, *c*_*i*_ denotes the output feature map, $\omega_{i}$ is the weight matrix, * denotes the convolutional product, *b*_*i*_ is the bias vector, and f(·) represents the activation function. The rectified linear unit (ReLU) function is commonly chosen as the activation function in CNNs. Mathematically, ReLU is defined as:


$$c_{i}=f(h_{i})=\max(0,h_{i}),$$

where *h*_*i*_ represents the elements of the feature map obtained from the convolution operation. Pooling operations reduce the size of feature maps by computing the maximum or average value within specified regions of the feature map, which helps prevent overfitting. Max pooling is a common pooling method, mathematically expressed as:


$$\gamma (c_{i},c_{i-1})=\max(c_{i},c_{i-1}),$$

$$p_{i}=\gamma (c_{i},c_{i-1})+\beta_{i},$$

where γ(·) represents the max pooling subsampling function, $\beta_{i}$ denotes the bias, and *p*_*i*_ indicates the output of the max pooling layer.

Finally, the feature maps obtained from the convolution and pooling operations are fed into a fully connected layer, which computes the final output vector as shown below,


$$y_{i}=f(t_{i}p_{i}+\delta_{i}),$$

where *y*_*i*_ represents the final output vector, $\delta_{i}$ is the bias term, and *t*_*i*_ is the weight matrix.

This configuration enables the CNN to effectively extract spatial features from the input sequence and feed them as input to the BiLSTM, thereby better capturing the temporal dependencies within the sequence data. This integrated model facilitates a more comprehensive understanding and learning of the inherent information in sequential data.

### 4.2 Bidirectional long short-term memory network

BiLSTM is a bidirectional structure that simultaneously extracts contextual information from both directions by utilizing forward and backward hidden layers [24]. The outputs $\vec{h}$ and $\overset{\to }{h}$ of the forward and backward layers are computed using standard LSTM units. The BiLSTM layer generates an output vector, where each element can be calculated using the following formula:


$$y_{t}=\delta ({\vec{h}}_{t}+{\overset{\to }{h}}_{t}),$$

where the function δ(·) is used to combine the two sequences $\vec{h}$ and $\overset{\to }{h}$ . The function δ(·) can be a summation, multiplication, concatenation, or averaging function. The final output of the BiLSTM layer can be represented as a vector *y*_*t*_ .

The framework of the CNN-BiLSTM method consists of two branches. One branch utilizes CNN to capture the attributes of financial indicators, while the other branch employs a two-layer BiLSTM for feature selection. Features from both branches are concatenated and fed into the final dense layer to generate the target predictions.

In the CNN branch, the input financial indicators *X*_*i*_ are passed through convolutional operators to capture underlying features of the raw data. Subsequently, max-pooling is performed to reduce the dimensionality of the feature maps. Dropout operations are utilized to prevent overfitting in CNNs. ReLU is used as the nonlinear activation function for this layer. Finally, a flattening layer is used to alter the dimensionality, followed by a dense layer for linear operations.

In the BiLSTM branch, a two-layer BiLSTM captures data information from the raw data. Each BiLSTM not only learns from the preceding point but also gains knowledge from the subsequent item in the current sequence, enabling the captured features to represent two different views of the *EDF* curve. The tanh function is selected as the nonlinear activation function in the BiLSTM layer. Features extracted from the BiLSTM are added to the output of the fully connected units. The outputs from the CNN branch and BiLSTM branch are merged and then fed into the final dense layer. The activation function of this dense layer is tanh. Finally, the CNN-BiLSTM model yields the predicted *y*_*t*_.

Specifically, this study utilizes 12 financial indicator data from listed agricultural enterprises, along with *EDF* calculated based on the KMV model, as input and output sequences to train the CNN-BiLSTM neural network. During training, optimizing network weights and minimizing errors are key objectives. After training, a well-trained CNN-BiLSTM neural network model is obtained. Subsequently, the financial indicators of non-listed agricultural enterprises are used as input sequences in the trained CNN-BiLSTM neural network to predict the corresponding *EDF*. This process effectively utilizes the previously trained network model to make predictions on new input data, thereby obtaining risk assessment of non-listed agricultural enterprises.

## 5 Empirical analysis

### 5.1 Data source and standardization

To predict *EDF* of non-listed agricultural enterprises, this study selects the annual equity and financial data of agricultural listed companies during 2022 for CNN-BiLSTM training. The selection criteria for data of listed agricultural enterprises in the training sample are determined based on aspects such as industry attributes and listing status. For non-listed agricultural enterprises, they are selected according to the principle of being smaller in scale and having a financial structure similar to that of listed enterprises. Abnormal data are handled by the elimination method, where samples with extreme values in the main variables are eliminated. Missing values are handled using the linear interpolation method, which reduces the deviation while retaining the sample information [19]. Training data are extracted from 105 A-share listed companies categorized under the Shenwan industry classification of agriculture, forestry, animal husbandry, and fishery, sourced from the Choice financial client. The data are used to determine corresponding *EDF* and are partitioned into training, validation, and test sets in proportions of 80%, 10%, and 10%, respectively.

To mitigate the impact of model features on the model itself, financial data are standardized using the Z-score normalization formula to bring them to the same scale. The standardized financial data are then input into the neural network for training. The formula for standardization is as follows:


$$x=(x-\bar{x})/\sigma ,$$

where $\bar{x}$ and *σ* represent the mean and the standard deviation of each column vector *x*, respectively. This normalization process ensures that each financial indicator is adjusted to have a mean of zero and a standard deviation of one. This aligns all features to a comparable scale for neural network training.

### 5.2 Construction of the CNN-BiLSTM neural network

**Fig 1:**
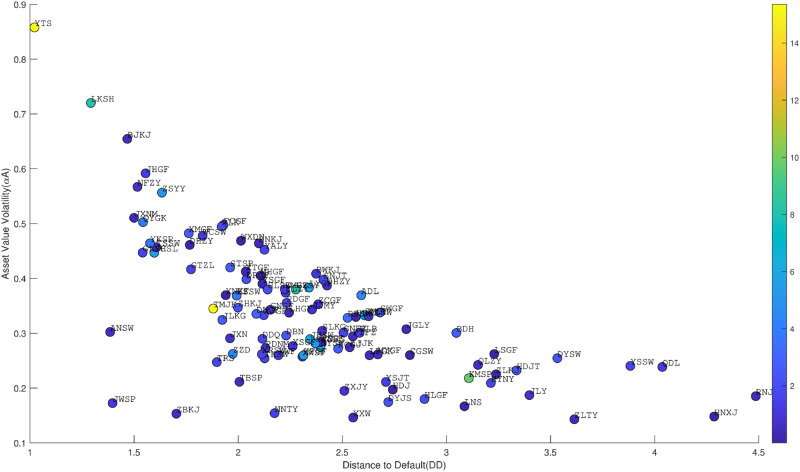
Scatter plot of EDF, DD, and asset value volatility.

We calculate the corresponding *EDF* based on the financial data of listed agricultural enterprises by using KMV model (5). Fig 1 presents the distribution heatmap of *EDF*, *DD*, and asset value volatility calculated for various agricultural enterprises. As shown in the figure, the computed *EDF* generally falls below 3%, indicating a good credit status for the corresponding enterprises.

The credit rating intervals are guided by Standard & Poor’s corresponding to *EDF* ranges of the KMV model. The threshold values of the rating range are set based on the data-driven principle [25]. They are divided according to the calculated EDF and the proportion of the six rating levels in the institution’s ratings. The calculated *EDF* for agricultural enterprises are then associated with corresponding rating intervals, and the descriptive statistics are presented in Table 2.

**Table 2:** Descriptive statistics of *EDF*(%) corresponding to rating intervals for agricultural enterprises.

Rating Interval	*EDF* Interval	Mean	Standard Deviation	Number of Enterprises
AA- and above	0-0.05	0.021	0.019	7
A- to AA-	0.05-0.14	0.081	0.024	4
BB+ to A-	0.14-0.86	0.56	0.192	28
B+ to BB+	0.86-2.88	1.7437	0.563	45
B- to B+	2.88-6.49	4.601	1.104	15
CCC+ and below	6.49-20	10.607	3.809	6

Based on Table 2 of calculated *EDF* and rating intervals of agricultural enterprises, the majority of agricultural enterprises exhibit low levels of *EDF*, indicating a favorable credit condition. Most enterprises are rated between B+ and A-, with relatively fewer enterprises rated A- and above or CCC+ and below. This suggests a concentrated distribution of credit ratings with a tendency towards the middle range and limited differentiation across rating levels.

### 5.3 Prediction model training of *EDF*

The model outputs the *EDF* of listed agricultural enterprises, calculated using the KMV model. The *EDF* is used as the output layer, with specific financial indicators of enterprises as input features. The learning rate is set to 0.001, with two hidden layers, each containing 12 neurons. The dataset is divided into training, validation, and testing sets in a ratio of 80%, 10%, and 10%, respectively. The training is set to run for 700 epochs.

### 5.4 Parameter settings and model comparison

To compare the model performance, we conduct a comparative analysis of prediction metrics from the CNN-BiLSTM model against other deep learning models. The models include: (a) long short-term memory (LSTM), (b) bidirectional long short-term memory (BiLSTM), (c) convolutional-LSTM (CNN-LSTM), (d) convolutional-bidirectional long short-term memory (CNN-BiLSTM), (e) convolutional gated recurrent unit (CNN-GRU), and (f) gated recurrent unit (GRU). Each model is applied to the data for prediction, and various evaluation metrics are calculated for comparison, as shown in Table 3.

**Table 3:** Statistical table of evaluation indicators for each model.

	MAE	MAPE	MSE	RMSE	*R*2
CNN-BiLSTM	0.1764	0.37094	0.052986	0.23019	0.99098
LSTM	0.76123	2.2534	1.3693	1.1702	0.76692
BiLSTM	0.59433	1.8957	0.69929	0.83624	0.88097
CNN-LSTM	0.21537	0.41436	0.085327	0.29211	0.98548
CNN-GRU	0.18162	0.47591	0.080857	0.28435	0.98624
GRU	0.84852	2.8443	2.286	1.5119	0.61088

In terms of model performance, the CNN-BiLSTM demonstrates exceptional results across various evaluation metrics. Specifically, it demonstrates excellent performance in terms of mean absolute error (MAE) and root mean square error (RMSE), which are 0.1764 and 0.23019, respectively. These values indicate a small deviation between predicted and actual values. Additionally, the CNN-BiLSTM model demonstrates significant advantages in mean squared error (MSE) and coefficient of determination (*R*2), with values of 0.1764 and 0.99098, respectively. This further confirms its excellent accuracy and explains variance in model predictions. The combined CNN and bidirectional long short-term memory model (CNN-BiLSTM) performs exceptionally well in predicting and evaluating sequence data. It is suitable for forecasting *EDF* of agricultural non-listed companies. In contrast, other models, such as LSTM and GRU, exhibit higher errors and lower coefficients of determination, reflecting relatively weaker performance in prediction tasks. Generally, non-listed enterprises have smaller scale and are less affected by fiscal and taxation as well as macroeconomic factors. The 12 financial indicators in Table 1 cover aspects such as profit and debt repayment, and mainly reflect the internal comprehensive financial situation. While listed enterprises, in addition to similar financial indicators, also involve capital market-related indicators such as stock price fluctuations and market value. Through Z-score standardization data, and with an *R*2 value of 0.99098, it has been verified that the differences in indicators do not significantly affect the applicability of the model.

**Fig 2:**
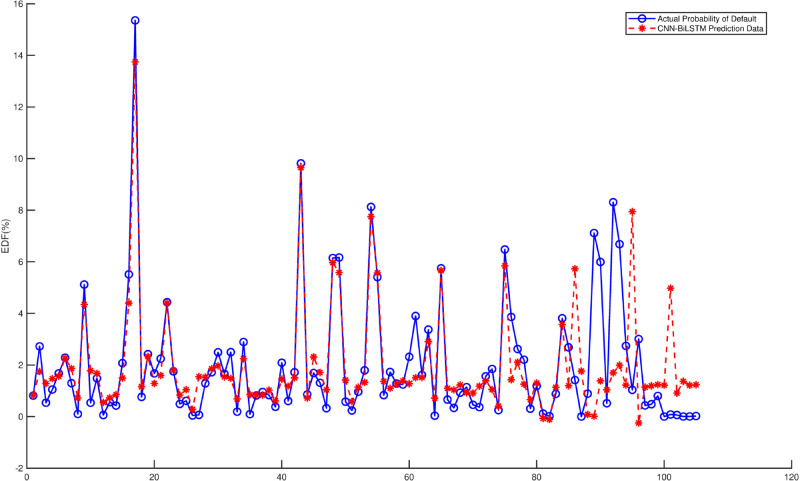
Comparison of CNN-BiLSTM predicted results with actual EDFs.

For the CNN-BiLSTM model, iterative training is conducted using MATLAB software, and the predicted results are compared with the actual data. From Fig 2, it can be observed that the predicted data generally fit well with the actual data, although there are a few instances where the data deviate from the actual situation. Therefore, applying the CNN-BiLSTM model to estimate *EDF* of an agricultural non-listed company is feasible. However, further optimization and adjustments are needed in practical applications to enhance the model’s predictive accuracy and robustness.

### 5.5 Optimization process of the CNN-BiLSTM model

**Fig 3:**
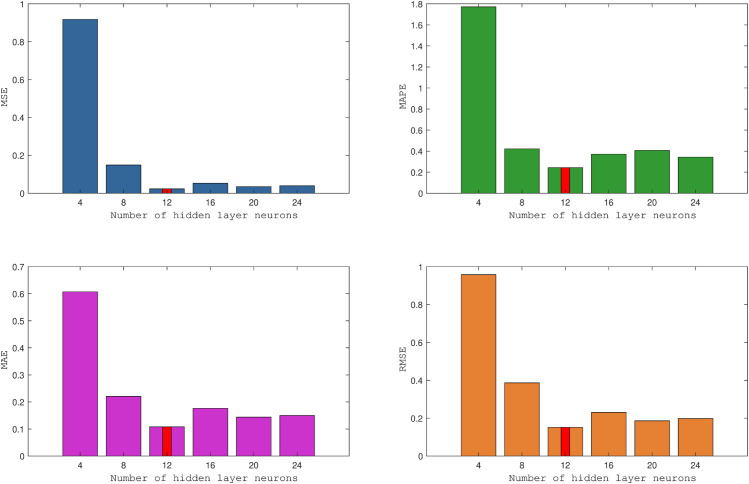
Comparison of evaluation metrics for number of hidden layer neurons in the LSTM model.

The number of hidden layer neurons in CNN-BiLSTM is a crucial parameter that impacts the model’s performance. A smaller number of neurons is suitable for smaller-scale networks with fewer parameters, making them easier to train and deploy quickly in models. Conversely, with larger datasets, a larger number of hidden layer neurons can typically be employed because more data supports more complex models. Increasing the number of hidden layer neurons, however, leads to increased computational complexity, requiring more computing resources and time for model training and deployment. In this study, we set the training epochs to 700 and varied the number of neurons per LSTM layer as 4, 8, 12, 16, 20, and 24, as shown in Table 4. Subsequently, we conduct comparative analyses of the results to assess the influence of varying numbers of hidden layer neurons on model performance. Please refer to Fig 3 for more details, which offers insights for selecting the optimal parameters.

**Table 4:** Comparison of error effects with different numbers of hidden layer neurons in the CNN-BiLSTM model.

Number of hidden layer	MSE	MAPE	MAE	RMSE	*R*2
4	0.91775	1.7718	0.60708	0.95799	0.84378
8	0.14914	0.42203	0.22057	0.38619	0.97461
12	0.022986	0.2431	0.10805	0.15161	0.99609
16	0.052986	0.37094	0.1764	0.23019	0.99098
20	0.034614	0.40691	0.14403	0.18605	0.99411
24	0.039173	0.34283	0.14983	0.19792	0.99333

In the CNN-BiLSTM model, optimal error feedback is observed when each LSTM layer has 12 hidden neurons. The selection of neuron count is an experimental and tuning process because the distribution of neurons and related optimizations is not directly linearly related. During training, all parameters are randomly generated. While a bar chart cannot directly reflect the trend of errors with changes in the number of neurons, we can conclude that the error is minimized when the neuron count is 12. Any increase or decrease beyond this value can lead to error amplification, thereby affecting the model’s predictive performance. Therefore, setting each layer of LSTM with 12 hidden neurons is the optimal choice in the experimental process.

### 5.6 Determination of training epochs for CNN-BiLSTM

The number of training epochs in the CNN-BiLSTM model typically has a significant impact on prediction results. Increasing the number of training epochs can enhance the model’s representational capacity, enabling it to better capture complex patterns and dependencies in the data. This is beneficial for solving intricate sequence data problems. With fewer training epochs, the model may not fully learn the data features, which can lead to the model not converging to its optimal performance level. Increasing the number of training epochs helps accelerate the model’s convergence rate and improve training efficiency. However, if the number of training epochs is too high, the model may overfit to the noise in the training data, thereby reducing its generalization ability.

As shown in Table 5, when the training epochs of the model are set to 700, the model demonstrates improved predictive performance with higher accuracy. When the number of training epochs exceeds 700, the model begins to show signs of overfitting, leading to a gradual decrease in predictive performance. Therefore, selecting 700 epochs as the training epochs for the CNN-BiLSTM model is considered appropriate.

**Table 5:** Comparison of error effects for different training epochs in CNN-BiLSTM model.

Training Epochs	MSE	MAPE	MAE	RMSE	*R*2
600	0.10882	0.31982	0.17023	0.32988	0.98148
700	0.022986	0.2431	0.10805	0.15161	0.99609
800	0.031416	0.25259	0.13081	0.17725	0.99465
900	0.054003	0.70242	0.17846	0.23239	0.99081
1000	0.054911	0.55939	0.17433	0.23433	0.99065

### 5.7 Credit rating

**Fig 4:**
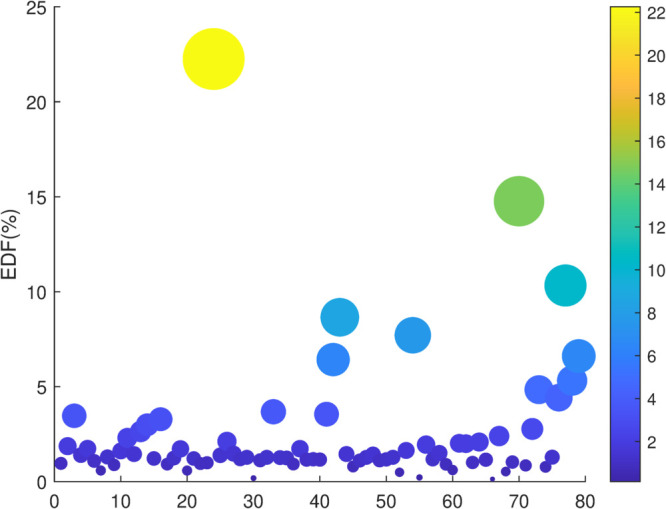
Predicted EDF for non-listed agricultural enterprises.

We calculate the *EDF* of various agricultural listed companies and use it to train an LSTM neural network. The neural network will be used to predict the measure of credit risk *EDF* of non-listed agricultural enterprises. The predicted *EDF* also serves as a reference for credit rating. We select financial data from 79 non-listed agricultural companies. The financial data is standardized using Z-scores, and is inputted into the trained CNN-BiLSTM model to predict *EDF*, as shown in Fig 4. In Fig 4, the majority of EDF of non-listed agricultural enterprises is below 3%, which is similar to the distribution trend of EDF of listed enterprises, reflecting the overall characteristic of credit risk in the agricultural industry [26].

Considering the restricted range of *EDF* for non-listed enterprises, we define three credit rating categories: high quality, medium quality and potential default. High quality corresponds to AAA and AA, along with their variations in institutional ratings, with *EDF* range of 0-2%. Medium quality is defined within the A to C in institutional ratings, with an *EDF* of 2%-4%.Rating with *EDF* exceeding 4% is categorized as potential default. Table 6 shows the number of credit ratings for non-listed agricultural companies.

**Table 6:** Number of credit ratings for non-listed agricultural enterprises.

	High quality	Medium quality	Potential default
Institutional rating	56	19	4
Our rating model	57	12	10

The results in Table 6 indicate that the number of unlisted agricultural enterprises rated as high quality obtained by our rating model is comparable to the number rated by institutions, and the proportion of consistent ratings is 98.25%. The number of enterprises rated as medium quality by our rating model is slightly less than that rated by institutions. In the potential default category, the number of enterprises rated by our model is 10, which is much larger than that rated by institutions. This reflects that our model has stricter rating criteria and a more accurate risk assessment. This also indicates that the distribution of sample EDF is consistent with the actual risk level of the industry and does not deviate from the distribution pattern of credit risk in agricultural enterprises.

## 6 Conclusions

By combining the BiLSTM neural network with the KMV model, this study utilizes the BiLSTM-KMV model to measure credit risk for non-listed agricultural enterprises. In the high quality category, which corresponds to an institutional rating of AA- and above, our model is basically consistent with the institutional ratings, with a consistency ratio of 98.25%. In the potential default category, our model demonstrates a more stringent rating standard. Some enterprises that perform as medium quality in institutional ratings may be rated as potential default. These discrepancies can stem from variations in rating criteria or limitations in the *EDF* scale.

The limitation of the model is that, although the credit ratings of non-listed agricultural enterprises by the model are generally consistent with the results of institutional ratings, there are occasional cases where the predicted data deviate from the actual expected default frequency. Further research can enhance the model’s adaptability to different market environments and enterprise types by expanding the time span and geographical scope of the training data.
